# Ectopic acromegaly with tumoral range hyperprolactinemia and apoplexy with a dramatic regression of pituitary hyperplasia

**DOI:** 10.3389/fendo.2024.1473167

**Published:** 2024-10-10

**Authors:** Ashish Gupta, Rajeev Kasaliwal, Liza Das, Surendra Kumar Sharma, Vaishali Kaur, Alexandre Vasiljevic, Véronique Raverot, Márta Korbonits, Pinaki Dutta

**Affiliations:** ^1^ Department of Endocrinology, Post Graduate Institute of Medical Education and Research (PGIMER), Chandigarh, India; ^2^ Department of Endocrinology, Mahatma Gandhi Medical College and Hospital, Jaipur, India; ^3^ Department of Telemedicine, Post Graduate Institute of Medical Education & Research (PGIMER), Chandigarh, India; ^4^ PraticienHospitalier, PharmacienBiologiste, Laboratoired’hormonologie, Hospices Civils de Lyon (Centre HospitalierUniversitaire de Lyon) CHU Lyon, Lyon, France; ^5^ Service d’Anatomie et CytologiePathologiques, Hospices Civils de Lyon (Centre Hospitalier Universitaire de Lyon), CHU Lyon, Lyon, France; ^6^ Department of Endocrinology, William Harvey Research Institute, Barts and the London School of Medicine, Queen Mary University of London, London, United Kingdom

**Keywords:** ectopic acromegaly, growth hormone-releasing hormone, pituitary hyperplasia, hyperprolactinemia, apoplexy

## Abstract

Acromegaly due to ectopic secretion of growth hormone-releasing hormone (GHRH) is a rare disorder. The signs and symptoms of ectopic acromegaly are indistinguishable from acromegaly due to a somatotroph adenoma. A 35-year-old female presented with secondary amenorrhea for 10 years, intermittent headache, and reduced vision in both eyes for 4 years, which worsened over 4 months before presentation. Additionally, she was diagnosed with uncontrolled diabetes mellitus. On examination, she had coarse facial features, a fleshy nose, and acral enlargement. She had diminished visual acuity (left>right) and bitemporal hemianopia on perimetry. Biochemical investigations revealed elevated IGF-1 [588 ng/ml, reference range (RR) 100–242], markedly elevated basal growth hormone (>80 ng/ml; RR, 0.12–9.88), and hyperprolactinemia in the tumoral range (832 ng/ml; RR, 5–25). MRI sella demonstrated a 22×30×34mm sellar-suprasellar mass with T2 hypointensity. Chest imaging revealed a 75×87×106mm left lung mass, which was found to be a well-differentiated neuroendocrine tumor (NET) on biopsy. Plasma GHRH levels were elevated [38,088 ng/l; RR, <250–300], and a diagnosis of ectopic acromegaly secondary to lung neuroendocrine tumor was considered. During workup, the patient developed in-hospital pituitary apoplexy, which improved with medical management. After a left pneumonectomy, her clinical features of acromegaly improved, her diabetes underwent remission, and there was a marked reduction in plasma GHRH and pituitary size. Histopathology was suggestive of a neuroendocrine tumor, with immunohistochemistry positive for GHRH and negative for prolactin. Her final diagnosis was ectopic acromegaly due to GHRH secreting a lung NET with pituitary somatotroph and lactotroph pituitary hyperplasia and apoplexy in the hyperplastic pituitary.

## Introduction

Acromegaly is a systemic disorder caused by growth hormone (GH) excess and is characterized by typical facial, acral, skeletal, and systemic manifestations affecting all the organ systems (1). Most often it occurs due to a GH-secreting tumor located in the sella, known as eutopic acromegaly. Ectopic acromegaly is extremely rare, comprising <1% of all acromegaly cases. The sources of ectopic growth hormone-releasing hormone (GHRH) include neuroendocrine tumors (NETs) arising from the pancreas, lung, thymus and appendix, pheochromocytomas, paragangliomas, and hypothalamic choristomas and gangliocytomas (2). Herein, we present a unique case of acromegaly due to a GHRH-secreting lung NET (bronchial carcinoid) causing enlargement of the pituitary gland, pituitary apoplexy, tumoral range hyperprolactinemia with remarkable resolution of clinical features hyperprolactinemia and IGF-1 levels after resection of the primary lung tumor.

## Case presentation

**Figure 1:**
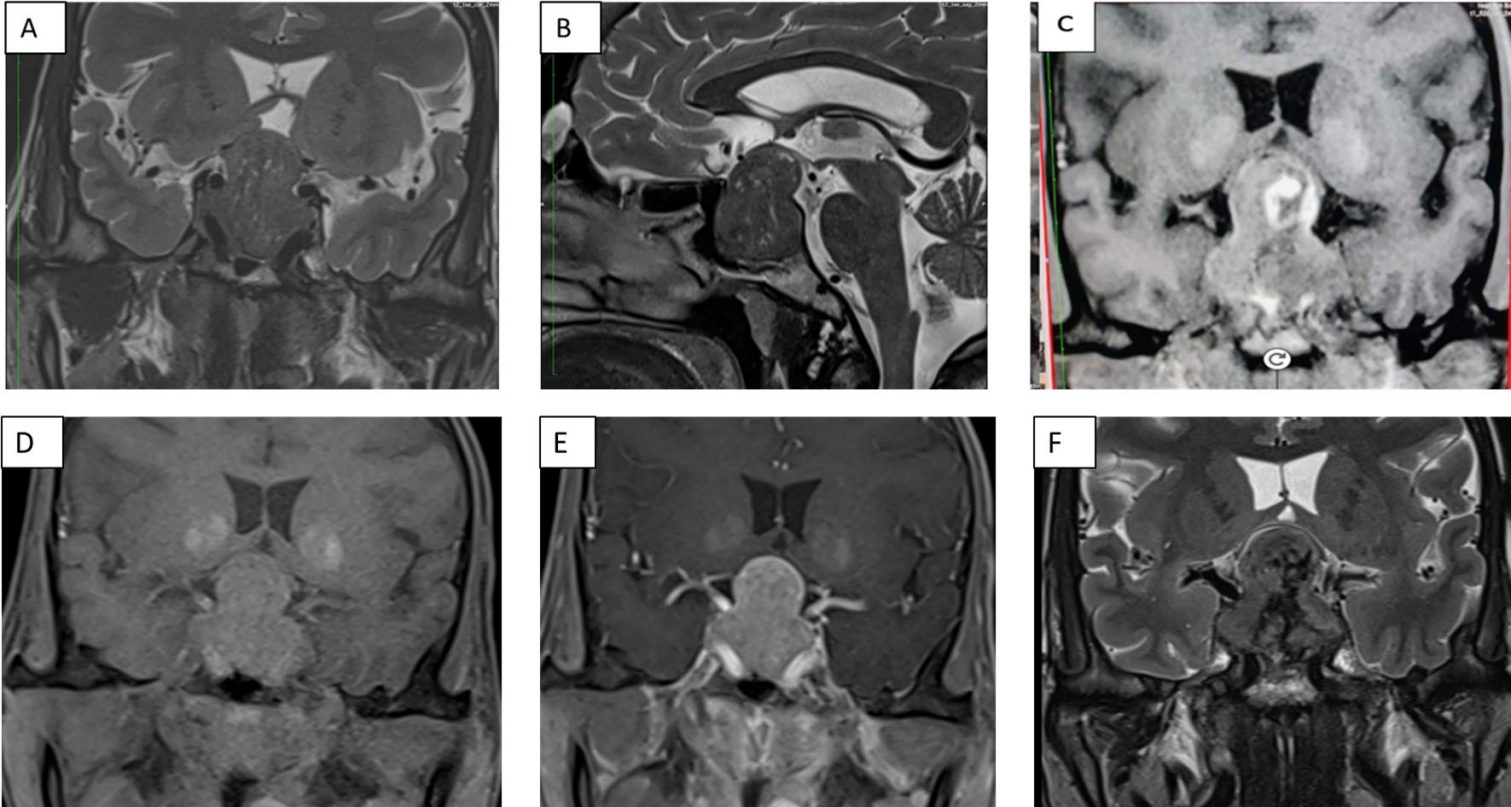
Panel of contrast-enhanced MRI of the sella showing **(A)** a 22×30×34 mm hypointense heterogenous lesion on a T2-weighted sequence coronal section **(A)** and sagittal section **(B)**, and coronal T1-weighted non-contrast **(D)** and post-contrast **(E)** sections. T1-weighted non-contrast **(C)** and T2-weighted **(F)** coronal sections show hyperintensities and hypointensities, respectively, with an increase in size of the lesion to 21×35×41mm, which is suggestive of pituitary apoplexy.

**Figure 2:**
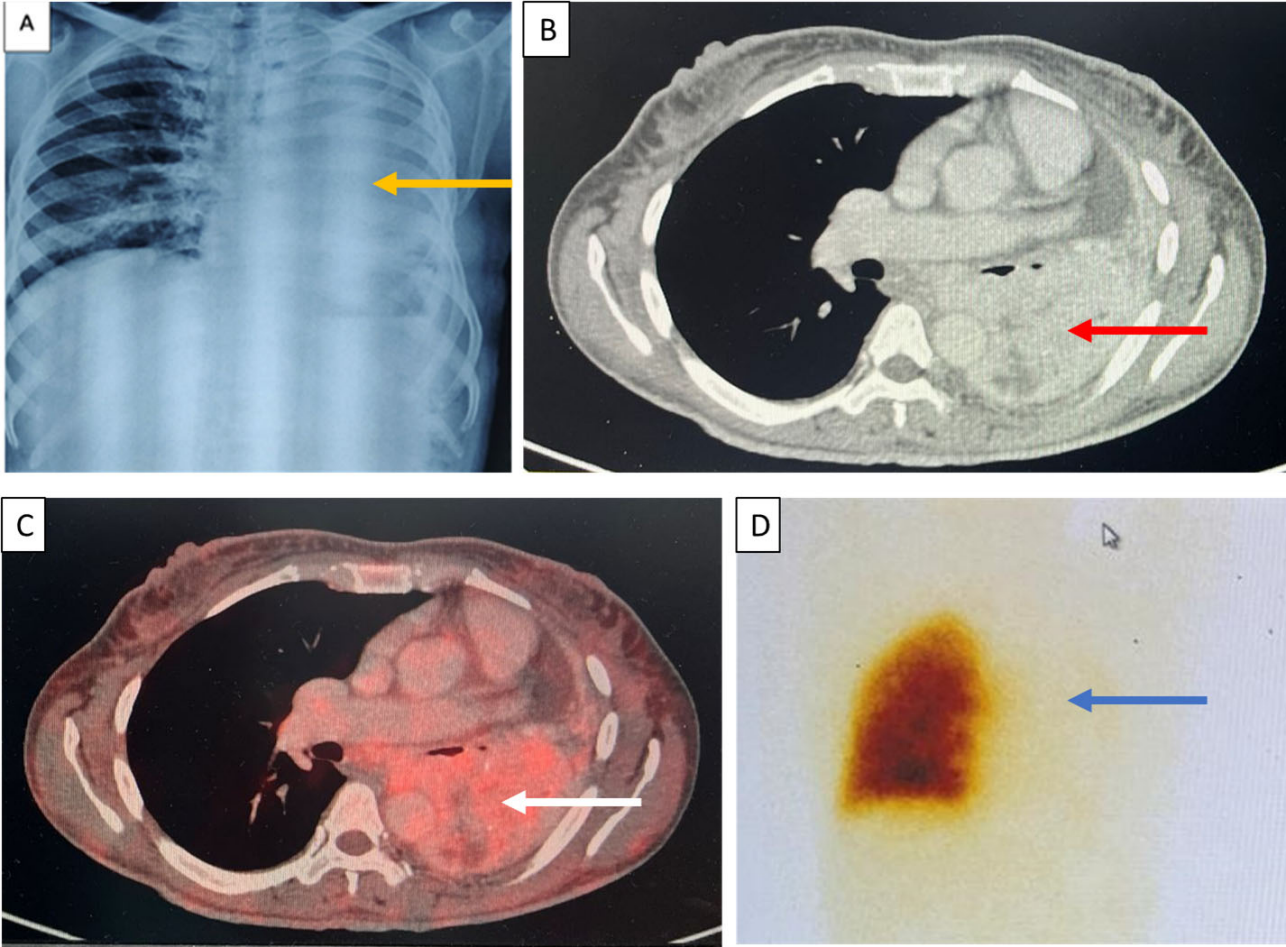
**(A)** Chest X-ray on day 4 of admission with complete opacification of the left hemithorax (yellow arrows). **(B)** Contrast-enhanced CT chest showing a heterogeneous mass with intense enhancement in the left lung (red arrow). **(C)** FDG-PET CT revealing an avid lung mass in the left lung (white arrow). **(D)** A ventilation perfusion scan suggestive of a severe decreased to negligible perfusion in the left lung (blue arrow).

A 35-year-old female presented with secondary amenorrhea for 10 years, intermittent headache, and reduced vision in both eyes for 4 years before presentation, which had worsened over the last 4 months. There was no history of galactorrhea, weight gain, striae, proximal muscle weakness, or other features suggestive of Cushing’s syndrome. She was diagnosed with diabetes mellitus 4 months before presentation to our hospital, with poor blood glucose control despite receiving four oral hypoglycemic agents [glimepiride (4 mg), metformin (1,000 mg), sitagliptin (100 mg), and dapagliflozin (10 mg) daily]. She had two children; she had breastfed the younger for 18 months 12 years ago. There was no personal or family history of pituitary or other tumors suggestive of multiple endocrine neoplasia type 1 (MEN1). On examination, her height was 140 cm (familial short stature), her BMI was 27kg/m², and she had coarse facial features, a fleshy nose, and acral enlargement. Her visual acuity was 2/60 in the right eye and 6/60 in the left eye. Fundus examination revealed bilateral moderate non-proliferative diabetic retinopathy (left eye> right eye). Visual field testing showed bitemporal hemianopia. Other aspects of systemic examination were normal. Her laboratory investigations are depicted in **Table 1**. She had raised baseline GH (>80 ng/ml) and IGF-1 [588 ng/ml; reference range (RR) 100–242]. She also had hyperprolactinemia [prolactin 330 and 832 ng/ml on two separate occasions (RR, 5–25ng/ml)]. Macroprolactin was normal. Further work-up revealed secondary hypocortisolism, hypogonadism, and hypothyroidism. Serum calcium (9.3 mg/dl; RR, 8.8–10.2) and phosphate (4.3 mg/dl; RR, 2.7–4.5) were normal. She was started on hormone replacement with daily prednisone (5 mg) and levothyroxine (100 mg). Contrast-enhanced dynamic MRI of the sella showed a 22×30×34mm sellar-suprasellar mass closely abutting the cavernous segment of the bilateral internal carotid artery and splaying of the optic chiasma, reported as pituitary macroadenoma (**Figure 1**). Initially, for headache and visual symptoms, she consulted a neurosurgeon and was scheduled for elective transsphenoidal excision for the pituitary lesion; however, as part of the pre-anesthetic check-up and evaluation of breathlessness, a chest X-ray was performed, which revealed a mass lesion in the left hemithorax (**Figure 2A**). Contrast-enhanced CT revealed a large heterogeneous mass in the left hilar region extending into the left upper lobe, lingula, and left lower lobe with an ipsilateral mediastinal shift (**Figure 2B**). The findings of the physical examination of the chest were missed by the treating doctors. Ultrasound-guided biopsy from the left lung mass revealed a well-differentiated NET with a Ki-67 of <2%, consistent with a WHO grade 1 tumor. Based on the above presentation and laboratory and radiological investigations, the possibility of an ectopic GHRH-secreting lung NET was raised. For further evaluation, an FDG PET scan was performed, which revealed avid lesions in the left lung (**Figure 2C**) and in the suprasellar region, without any other uptake elsewhere. Her arterial blood gas analysis was within normal limits, but the ventilation-perfusion scan suggested a severely decreased perfusion in the left lung (**Figure 2D**). Plasma GHRH levels, measured using a radioimmunoassay, as previously described (3), were extremely high (38,088 ng/l; RR <250–300 ng/l).

**Table 1:** Pre- and post-operative biochemical and hormonal investigations in the patient.

Biochemical parameter (normal range)	Pre-operative	3 months postoperative	9 months postoperative
IGF-1 (100-242 ng/ml)	588	137	124
GH (0.12-9.88 ng/ml)	>80	0.54	NA
GHRH (<250-300 ng/l)	38,088	458	NA
Prolactin (5-25 ng/ml)	330, 832	NA	3.6
FSH (2.9-9 mIU/ml)	0.53	NA	1.41
LH (2-8 mIU/ml)	0.1	NA	0.41
HbA1C (%)	10.5	5.7	4.9
fT3 (2.77-5.27 pg/ml)	3.39	2.23	2.79
fT4 (0.78-2.19 ng/dl)	0.71	1.1	0.98
TSH (0.46-4.68 µIU/ml	2.215	0.4	0.53
Cortisol (1.7-14 µg/dl)	4.66	0.86	0.19
iPTH (7.7-53.5 pg/ml)	56	NA	106
Calcium (8.4-10.2 mg/dl)	9.3	NA	9.4
Inorganic phosphate (2.5-4.5 mg/dl)	4.3	NA	4.4
Alkaline phosphatase (38-126 IU/l)	96	NA	64

NA, not available.

**Figure 3:**
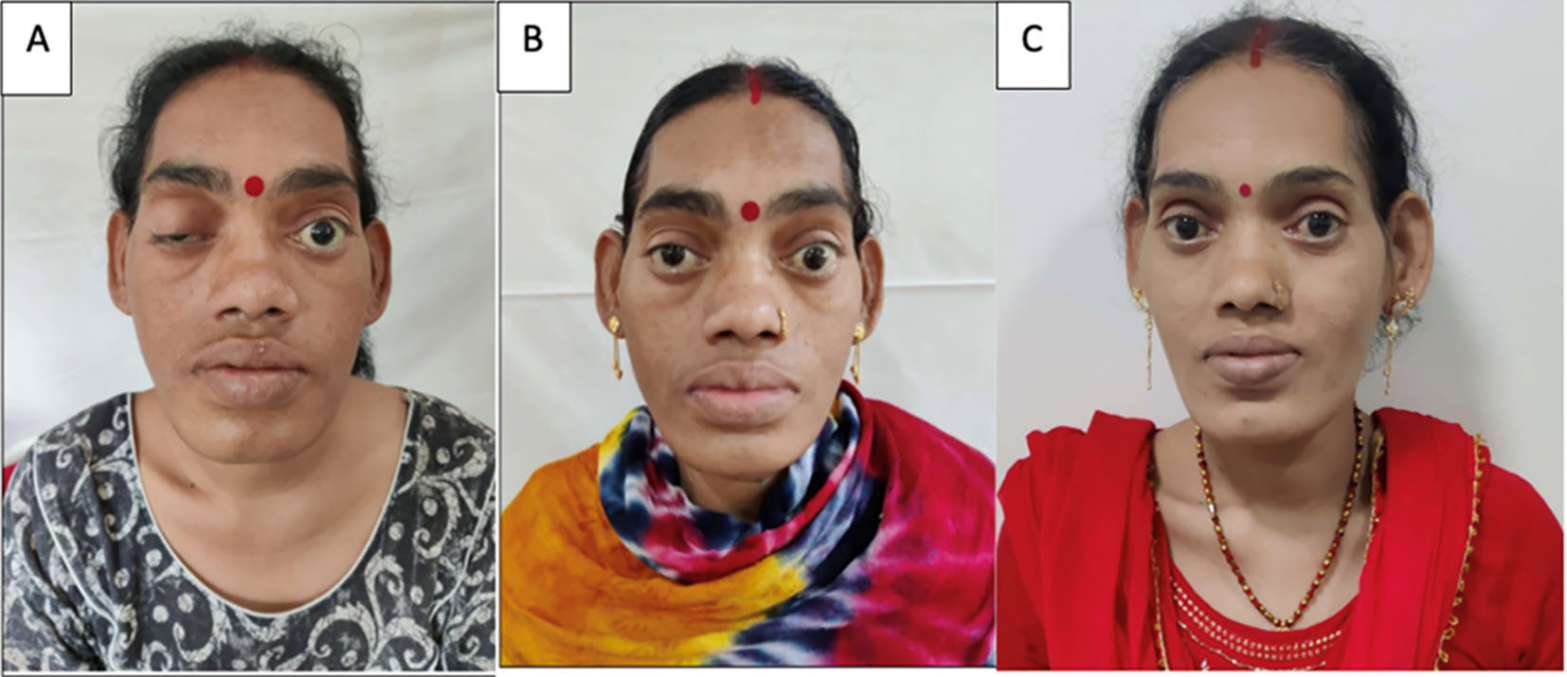
Clinical photographs of the patient. **(A)** At presentation showing ptosis of the right eye and coarse facial features, **(B)** resolution of the ptosis with persistent coarse features after 5 days with conservative management of apoplexy, and **(C)** an image after 9 months showing the resolution of the coarse facial features after surgical excision of the lung mass.

**Figure 4:**
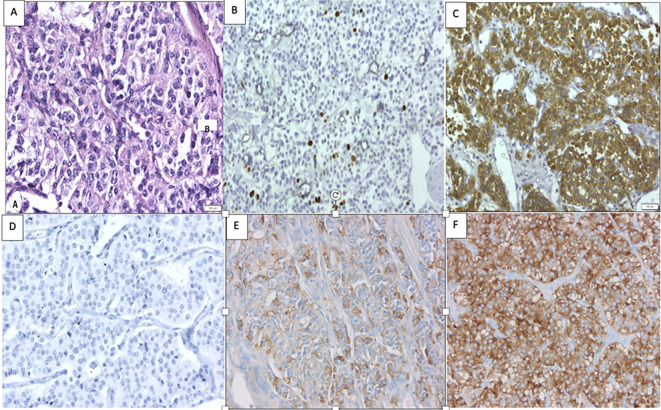
Histopathology of the lung mass with **(A)** hematoxylin and eosin staining revealing features of lung NET (400×). **(B)** A Ki-67 index of <3% (400×). **(C)** Positive for synaptophysin (400×). **(D)** Negative immunohistochemistry for prolactin negative (400×). **(E)** Immunohistochemistry for GHRH demonstrating focal positivity (5% of cells) (400×). **(F)** Positive GHRH control of a previously studied lung NET (400×).

**Figure 5:**
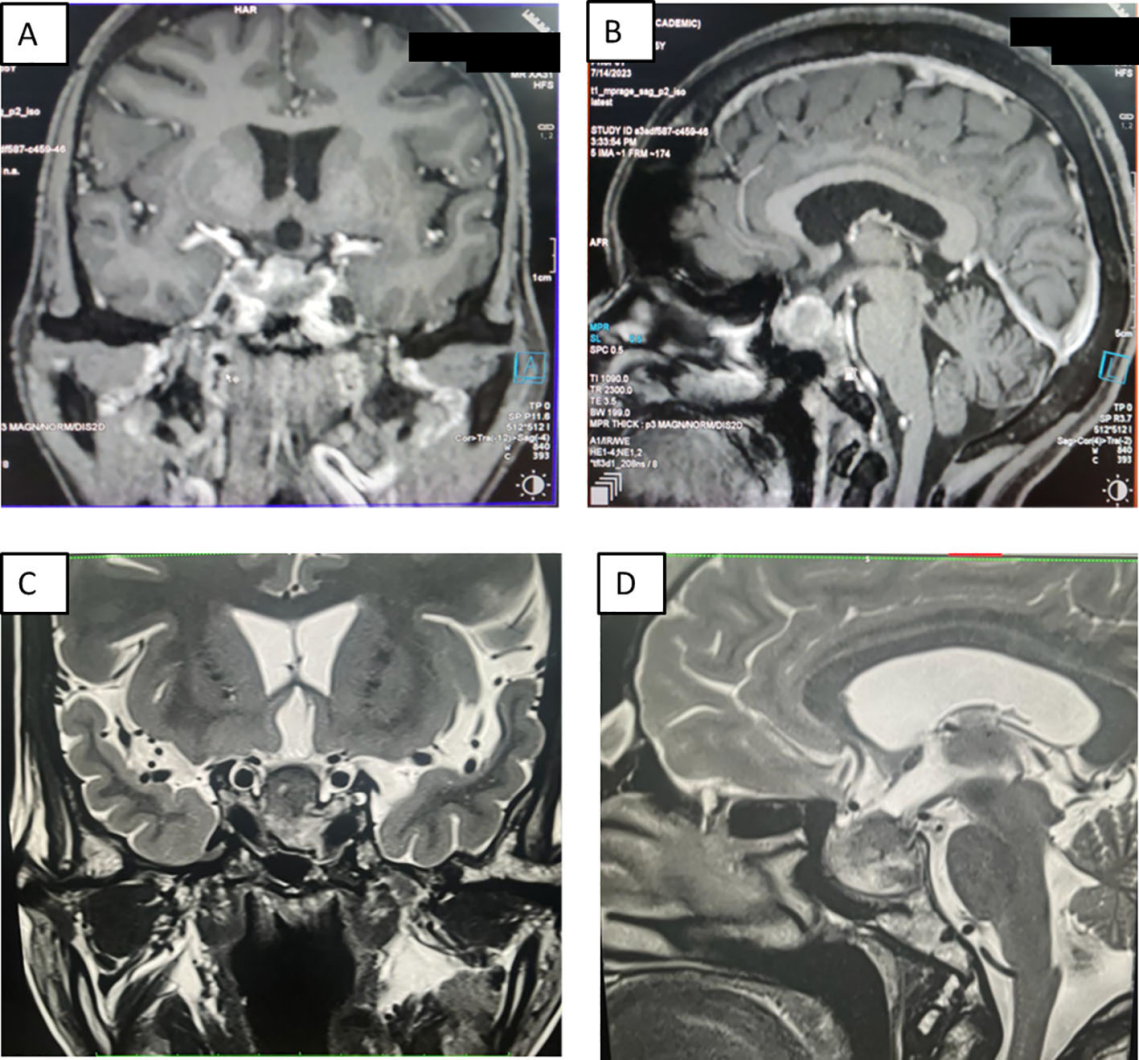
Panel of a contrast-enhanced MRI of the sella showing the initial reduction in size of a residual lesion to 20×24×24 mm (48% reduction) 3 months after lung surgery on coronal **(A)** and sagittal sections **(B)**. Further significant reduction of the lesion to 19×20×17.6 mm (70% reduction) in the coronal **(C)** and sagittal **(D)** sections 9 months after lung surgery.

While awaiting definitive management in the hospital, the patient developed severe headaches, multiple episodes of vomiting, drooping of the right eye (**Figure 3A**), and a deterioration of visual acuity (only perception of light in the right eye and finger counting at 2 m with a best corrected visual acuity of 6/60 in the left eye). MRI revealed apoplexy with an increase in size of the sellar-suprasellar mass (**Figure 1C**). The patient was managed with intravenous hydrocortisone (100 mg) every 8 h for 5 days followed by oral prednisone (7.5 mg) daily. Short-acting octreotide (100 mcg) administered subcutaneously every 8 h was initiated. After an improvement in her general condition, she underwent a left pneumonectomy a week later, and octreotide was discontinued. During surgery, a large yellow-colored tumor was identified. Her headache and ptosis improved within 5 days of the pneumonectomy (**Figure 3B**) and GH decreased to 22.4 ng/ml and prolactin decreased to 1.9 ng/ml. Histopathology revealed a NET with a Ki-67 of <3%, and immunohistochemistry was positive for synaptophysin, thyroid transcription factor 1 (TTF-1), and GHRH. Immunohistochemical analysis was negative for GH and prolactin (**Figures 4A–D**). The visual fields and acuity improved at 3 months (6/12 in the left eye and finger counting at 3 m in the right eye) along with a regression of soft tissue enlargement (**Figure 3C**). Repeat biochemical assessment at 3 months showed a normal IGF-1 (133 ng/ml; RR, 100–242) and GH (0.54 ng/ml), and a marked decrease in GHRH (458 ng/l; RR <250–300) (**Table 1**). Pituitary MRI at 3 months (**Figure 5A, B**) revealed a reduction in the size of the sellar-suprasellar mass by 48.7% (20×24×24mm). Whole- exome sequencing of peripheral blood-derived DNA showed no pathogenic variants in the *MEN1*, *CDKN1B*, and *AIP* genes. She continued to receive oral glucocorticoids and levothyroxine for central hypocortisolism and hypothyroidism. At 9 months follow-up, she was asymptomatic (**Table 1**), with significantly improved visual acuity (6/60 in the right eye and 6/12 in the left eye) and a resumption of spontaneous menstrual cycles. There was a further decrease in the size of the pituitary mass to 19×20x×17.6mm (**Figures 5C, D**), consistent with a 70.2% reduction in tumor volume.

## Discussion

Here, we present a case of ectopic acromegaly caused by a GHRH-secreting lung NET. The unique features of the case include marked hyperplasia of the pituitary gland mimicking pituitary macroadenoma, elevated prolactin in the range usually seen in macroprolactinomas, and apoplexy in the hyperplastic pituitary gland. There was a dramatic regression of facial features and pituitary lesion volume within 3 months after the removal of the lung NET, with near-normal GHRH and prolactin levels, implicating that the GHRH-secreting lung tumor caused the acromegaly.

Ectopic acromegaly contributes to <1% of all cases of acromegaly. The clinical characteristics of ectopic acromegaly are indistinguishable from those of acromegaly resulting from GH-secreting pituitary adenomas. Lung NETs are the most common cause of ectopic acromegaly, accounting for 43% of all cases, followed by pancreatic NETs, which account for 35% of cases (2). Females are more commonly affected than males (70.1% vs. 29.9%) (2).

GHRH has been shown to be highly specific for ectopic acromegaly, as shown in a series of 177 consecutive GH-secreting adenomas in which all patients had undetectable plasma GHRH. A GHRH cutoff of 250–300 ng/l has been proposed to have a high specificity (93.8%) for the diagnosis of ectopic acromegaly, along with imaging (4, 5). However, it is not known whether GHRH levels between 30–250 ng/l are physiological or can be due to mild GHRH excess. GHRH is expressed in a variety of normal peripheral tissues, including the gastrointestinal tract, lymphocytes, uterus, ovary, testis, placenta, cerebral cortex, pituitary, kidney, prostate, liver, and lung. The major contributor to plasma GHRH is the gastrointestinal tract and the sample for GHRH needs to be taken in a fasting state (6). Our case had unequivocally high GHRH levels pre-operatively (38,088 ng/l), which decreased post-operatively to 458 ng/l (<250–300 ng/l). This was slightly above normal and could be attributed to microscopic lymph node metastases (as seen in two of four resected lymph nodes), circulating bioinactive GHRH, or secretion from other physiological sources of GHRH, such as the gastrointestinal tract, lymphocytes, uterus, ovary, cerebral cortex, pituitary, kidney, liver, or lung. A contrast-enhanced CT carried out at 9 months did not reveal any macroscopic residue, and her GH and IGF1 values were normal until the reporting of this case. Nevertheless, because of a detectable GHRH value in the post-operative period she was kept under constant surveillance to look for a residue or recurrence.

An interesting feature in this case was the markedly elevated prolactin at diagnosis, in a range that is only seen in patients with macroprolactinoma. Ectopic prolactin secretion from the lung lesion was excluded by a negative immunohistochemical analysis for prolactin in the lung NET. Ectopic prolactin production has been reported from tumors such as a leiomyoma of the uterus, a gonadoblastoma, an ovarian teratoma, a perivascular epithelioid cell tumor, a uterine cervical carcinoma, and a colorectal adenocarcinoma (7). Elevated serum prolactin was reported in 35% (44/124) or 29% (6/21) of cases in reviews of ectopic GHRH secretion (**Table 2**). Our case is unique in that prolactin levels were markedly elevated pre-operatively and decreased to normal levels after 9 months of follow-up (**Table 1**). Hyperprolactinemia is likely attributable to increased GHRH, which is known to cause high prolactin in normal subjects and in patients with acromegaly, due to lactotroph hyperplasia (15, 16). In stalk disinhibition syndrome, prolactin levels seldom exceed 100 ng/ml (17). Marked prolactin elevation should also lead to a suspicion of MEN1 syndrome (18). However, in our case, it was excluded due to a lack of clinical signs and characteristic family history and by genetics analysis.

**Table 2:** Literature review of cases with hyperprolactinemia associated with ectopic acromegaly due to GHRH secretion.

Case no.	Age (years)	Gender	Amenorrhea	Galactorrhea	Acral enlargement	Prolactin baseline	GH baseline	GH nadir on OGTT	IGF-1 baseline	GHRH	Pre-operative radiology (MRI/CT of sella)	Sellar volume	T2 intensity on MRI	Location of primary tumor	MEN-1 mutation	HPE of pituitary	IHC for prolactin	HPE of primary tumor	IHC for GHRH	Prolactinpost-operative
Case 1 (8)	33	F	present	present	present	1040mU/l (82-368), 48.8 ng/ml	3.9mU/l (1.2 ng/ml)	12·7mU/l (4.2 ng/ml)	196nmol/l (14–41) (1499 ng/ml)	193pg/l (normal value < 78 pg/l)	Normal	NA	NA	Thymus	Positive	NA	NA	Thymic carcinoid with vascular invasion and lymph node metastases	+	1060mU/l(49.8 µg/l)
Case 2 (9)	21	F	Present (Turner syndrome)	present	present	88ng/ml	151 ng/ml	111ng/ml	11U/ml (normal <2)	NA	Hyperplasia	1,440 mm3(242- 1,042)	NA	Pancreas	NA	Normal pituitary	positive	Pancreatic NET	NA	22ng/ml
Case 3 (10)	27	F	present	present	present	20ng/ml (ULN: 12 ng/ml)	99 mU/l (32.97ng/ml)	55mU/l(18.3 ng/ml)	86nmol/l(ULN: 32 nmol/l)	2,519pg/ml (<50 pg/ml ULN)	Hyperplasia	NA	NA	Lung	NA	NA	NA	Lung NET	NA	31–42mU/l(1.5-2.0 ng/ml)
Case 4 (10)	27	M	–	absent	present	21 ng/ml (ULN: 6 ng/ml)	110 mU/l(36.63ng/ml)	52mU/l(17.3 ng/ml)	63nmol/l(ULN: 32 nmol/l)	49000pg/ml	Macroadenoma	NA	NA	Lung	NA	Hyperplasia and adenoma	positive	Lung NET	NA	NA
Case 5 (11)	23	F	present	present	present	45-48ng/ml	16 to 67 ng/ml	38 to 41ng/ml	NA	NA	Increased pituitary height 10 mm (CT)	NA	NA	Jejunum	NA	NA	NA	Jejunal carcinoid	+	18ng/ml(18 months post-op
Case 6 (12)	28	F	present	present	present	1230 mU/l (ULN < 425 mU/l), 57.8ng/ml	35 to 43 ng/ml	NA	NA	NA	Enlargement	NA	NA	Lung	NA	NA	NA	Bronchial carcinoid	+	261mU/l, 355 mU/l(12.3, 16.7 ng/ml)
Case 7 (13)	46	F	present	present	present	33 ng/ml (<26 g/ml)	43 ng/ml	NA	1054ng/ml (reference range, 49-292 ng/ml)	>1000pg/ml(<18 pg/ml)	Sellar mass 1.2 cm size	NA	NA	Pancreas	Positive	Adenoma with focal prolactin positivity and surrounding pituitary hyperplasia GH positive	positive	Pancreatic NET	+	NA
Case 8** (14)	53	F	not available	absent	present	~10ng/ml at baseline and maximum 70 ng/ml during follow up	NA	15ng/ml	768ng/ml(81-225 ng/ml)	8315ng/l at diagnosis	Enlarged pituitary15.7 mm in transverse diameter.	NA	hypointense	Lung	NA	NA	NA	Bronchial carcinoid	NA	Medical management offered. NA
Case 9 (5)	35	F	NA	NA	NA	Elevated (value not available)	40 ng/ml	NA	NA	2600 ng/l	Macroadenoma 11mm	NA	NA	Pancreas	Positive	NA	NA	WDET	NA	NA
Case 10 (5)	14	M		NA	NA	31ng/ml	57 ng/ml	27ng/ml	3.3 ULN	376ng/l	Macroadenoma 20 mm	NA	NA	Pancreas	Positive	Hyperplasia	NA	WDEC	NA	NA
Case 11 (5)	27	F	NA	NA	NA	35ng/ml	94ng/ml	118 ng/ml	3.8 ULN	548ng/l	Enlargement	NA	NA	Pancreas	NA	NA	NA	WDEC	NA	NA
Case 12 (5)	34	F	NA	NA	NA	45ng/ml	43ng/ml	30ng/ml	3.4 ULN	512ng/l	Macroadenoma	NA	NA	Pancreas	Positive	Hyperplasia and adenoma	positive	WDEC	NA	NA
Case 13 (5)	47	M		NA	NA	Elevated (value not available)	NA	NA	3.3 ULN	721ng/l	Adenoma (size not specified)	NA	NA	Pancreas	Positive	Hyperplasia and adenoma	positive	NP	NA	NA
Case 14 (5)	17	M		NA	NA	256ng/ml	256ng/ml	156ng/ml	1.3 ULN	534 ng/l	Macroadenoma 20 mm	NA	NA	Pancreas	Positive	NA	NA	WDEC	NA	NA
Current Case	35	F	present	present	present	832ng/ml (5-25)	>80 ng/ml	NA	588ng/ml (74-196)	38,088ng/L (<250-300)	22x30x34 mm pituitary macroadenoma	11,754mm3	hypointense	Lung	Negative	NA	NA	Bronchial carcinoid	+	3.6ng/ml (5-25)

*M, Male; F, Female; ULN, Upper limit of normal; IHC, immunohistochemistry; OGTT, Oral Glucose Tolerance Test; WDEC, well-differentiated endocrine carcinoma; WDET, well-differentiated endocrine tumor; NP, not performed.

**In this case, prolactin was normal at baseline and increased on subsequent follow-up, suggesting that a prolonged trophic effect of GHRH on lactotropes is required.

Pituitary hyperplasia can be mistaken for pituitary macroadenoma on imaging. In a large review of ectopic GHRH secretion with acromegaly, 43 out of 96 cases were reported as pituitary enlargement, 20 as adenomas, 18 normal on imaging, 10 as unclear lesions, 3 as empty sellae, and 2 as microcystic lesions (2). Similarly, in another retrospective study comprising 30 cases of GHRH-secreting neuroendocrine tumors, radiological hyperplasia of the pituitary was reported in 80% (24/30) of cases (20). However, on histopathology, only 20/29 were hyperplasia, 3/29 cases turned out to be adenoma, 3/29 mixed adenoma, and hyperplasia, 2/29 cases there in no mention of histopathological finding and 1 out of 29 normal pituitary was found (2). In another series of 21 cases, 5/21 cases of ectopic acromegaly due to GHRH with hyperprolactinemia were reported to have adenoma on imaging (4 macroadenoma and 1 adenoma not specified) (5). Three of these cases underwent pituitary surgery, with histopathology revealing somatotroph hyperplasia in one and somatotroph hyperplasia with prolactin-GH adenoma in the other two cases (5). All the patients had ectopic acromegaly resulting from gastro-pancreatic NETs and four out of these six cases had metastases at diagnosis, mainly to the liver. Our case, however, had a lung NET and the sellar-suprasellar mass was 34 mm in maximum dimension, with the radiologist reporting it as a pituitary macroadenoma. Adenoma can be seen in areas adjacent to hyperplasia. It has been demonstrated that excessive GHRH stimulation leads to somatotroph hyperplasia and, ultimately, pituitary adenoma formation in metallothionein promoter-driven human GHRH transgenic mice (19), some of which had shown focal positivity for prolactin, further emphasizing the phenomenon of specificity spillover akin to thyro-lactotroph hyperplasia in long-standing untreated juvenile hypothyroidism (20). A characteristic radiological feature attributed to ectopic acromegaly is the presence of T2-weighted hypointensity on MRI, which was present in 83% (25/30) of cases in a previous series. Rarely, microcysts (2.1% to 9.5% in some series) have been reported (21). Although T2 hypointense signal may be present in eutopic acromegaly as well, the prevalence is much lower (52.9%), as observed in a large series of 297 patients (22).

Histopathologically, hyperplasia was present in 20, adenoma in 3, and both adenoma with surrounding areas of hyperplasia in 3, unrelated lesions in 2, and not specified for 1 respectively. The exact histopathological nature of the lesion in our patient is unknown, as she did not have pituitary surgery. Another interesting feature of the current case was the apoplexy in the pituitary hyperplasia that occurred during the course of the hospital stay. The patient presented with right eye ptosis, which improved significantly over 5 days with conservative management. The index patient had a large sellar suprasellar mass, which was possibly the only predisposition for apoplexy. She did not have any other known risk factors such as hemodynamic disturbances due to major surgery, an intervention for surgery or gamma knife radiosurgery, hypertension, a hemorrhagic pregnancy, anticoagulation use, endocrine function tests (stimulation tests), cabergoline use, or a vasculotoxic snake bite. Although histopathological analysis of the sellar-suprasellar mass was not available in the current case, the rapidity of volume reduction after removal of the primary tumor favors a diagnosis of hyperplasia over adenoma. Apoplexy has been reported in hyperplasia, with only a single case in the past in which histopathology of the pituitary revealed an adenoma adjacent to hyperplasia (19).

Despite her history of long-standing secondary amenorrhea and exertional dyspnea for the last 3 months in retrospect, her lung mass could be diagnosed only during a pre-operative assessment before pituitary surgery. The pre-operative radiological impression was that of an invasive macroadenoma that could be a hyperplasia, as the size decreased significantly and dramatically after the removal of the primary tumor, with a dramatic improvement in signs and symptoms. Moreover, histopathology also revealed positive GHRH immunostaining in the lung tissue, with a decrease in serum GHRH levels post-surgery.

Regarding the prognosis of GHRH-secreting tumors (NETs), surgical resection of the primary tumor is the treatment of choice in the majority of cases. The prognosis of these patients is excellent, with survival exceeding 80% in the majority of cases and up to 94% in lung tumors, as described previously (2, 23). This is despite the fact that up to 50% of these patients can have metastatic disease at diagnosis. Residual disease following surgery can be managed successfully by using somatostatin-receptor ligands (SRLs) or, rarely, even pegvisomant. Adjuvant medical management can lead to the successful resolution of symptoms and normalization of IGF1, with or without a significant decrease in GHRH (24).

## Conclusion

Pituitary hyperplasia due to ectopic GHRH-secreting tumors can radiologically mimic pituitary adenoma, and apoplexy is extremely unusual but can lead to rapid shrinkage of the tumor, unlike eutopic somatotropinomas. Hyperprolactinemia is not uncommon but is rarely in the tumoral range in GHRH-secreting ectopic acromegaly.

## Data Availability

The original contributions presented in the study are included in the article/Supplementary Material. Further inquiries can be directed to the corresponding author.
